# Highly efficient mesophyll protoplast isolation and PEG-mediated transient gene expression for rapid and large-scale gene characterization in cassava (*Manihot esculenta* Crantz)

**DOI:** 10.1186/s12896-017-0349-2

**Published:** 2017-03-14

**Authors:** Jun-Zheng Wu, Qin Liu, Xiao-Shan Geng, Kai-Mian Li, Li-Juan Luo, Jin-Ping Liu

**Affiliations:** 1 Hainan Key Laboratory for Sustainable Utilization of Tropical Bioresources, College of Agriculture, Hainan University, Haikou, Hainan Province 570228 China; 2 The Institute of Tropical Bioscience and Biotechnology, Chinese Academy of Tropical Agricultural Sciences, Haikou, Hainan Province 571101 China

**Keywords:** Cassava, *Manihot esculenta*, Protoplast isolation, Transient expression system, Subcellular localization

## Abstract

**Background:**

Cassava (*Manihot esculenta* Crantz) is a major crop extensively cultivated in the tropics as both an important source of calories and a promising source for biofuel production. Although stable gene expression have been used for transgenic breeding and gene function study, a quick, easy and large-scale transformation platform has been in urgent need for gene functional characterization, especially after the cassava full genome was sequenced.

**Methods:**

Fully expanded leaves from in vitro plantlets of *Manihot esculenta* were used to optimize the concentrations of cellulase R-10 and macerozyme R-10 for obtaining protoplasts with the highest yield and viability. Then, the optimum conditions (PEG4000 concentration and transfection time) were determined for cassava protoplast transient gene expression. In addition, the reliability of the established protocol was confirmed for subcellular protein localization.

**Results:**

In this work we optimized the main influencing factors and developed an efficient mesophyll protoplast isolation and PEG-mediated transient gene expression in cassava. The suitable enzyme digestion system was established with the combination of 1.6% cellulase R-10 and 0.8% macerozyme R-10 for 16 h of digestion in the dark at 25 °C, resulting in the high yield (4.4 × 10^7^ protoplasts/g FW) and vitality (92.6%) of mesophyll protoplasts. The maximum transfection efficiency (70.8%) was obtained with the incubation of the protoplasts/vector DNA mixture with 25% PEG4000 for 10 min. We validated the applicability of the system for studying the subcellular localization of MeSTP7 (an H^+^/monosaccharide cotransporter) with our transient expression protocol and a heterologous *Arabidopsis* transient gene expression system.

**Conclusion:**

We optimized the main influencing factors and developed an efficient mesophyll protoplast isolation and transient gene expression in cassava, which will facilitate large-scale characterization of genes and pathways in cassava.

## Background

Transient gene expression in plant protoplasts is a powerful technique for studying subcellular localization of proteins, gene and promoter activities, protein-protein interactions, and signal transduction [1–7]. Compared with the stable gene expression in transgenic plants, transient gene expression represents a fast, convenient and efficient alternative system with higher expression levels [8–10]. Moreover, the transient gene expression assay enable the high-throughput analysis of gene functions while the stable gene expression is relatively expensive and time-consuming thus limiting the utilization of this technique for large-scale analyses of plant genes [8–10]. Although different transfection techniques such as polyethylene glycol (PEG)-mediated, electroporation and microinjection have been developed to deliver recombinant DNA plasmids into protoplasts, utilization of the PEG-mediated approach has high transformation efficiency, so it is widely applied in molecular and cellular studies for both model plants and non-model plants [4, 7, 8, 11–20].

Cassava (*Manihot esculenta* Crantz) is a perennial and woody shrub of the Euphorbiaceae cultivated in tropical and subtropical regions for its starchy storage roots [21, 22]. These roots have been used as the sources for dietary carbohydrate, starch processing and potential biofuel production. Since that cassava is a staple crop for approximately 800 million people in developing regions of the tropics [23], many groups have been intensively performing research on cassava molecular breeding which relies on the identification of agronomically important genes and pathways. Recently, the cassava sequencing data set has been publicly released [24] and the determination of gene function has been a major goal of cassava molecular biology in genomic and post-genomic era, thus it will necessitate efficient high-throughput transient gene expression for gene function analysis.

Stable gene transfer can be developed for gene function studies at whole plant level. Much progress has been made in cassava genetic transformation of both *Agrobacterium tumefaciens*-mediated and biolistic-mediated system [23, 25–27]. However, the transformation efficiency is relatively low and genotypic dependent [28], and the process requires well-trained tissue culture specialists, is lengthy and difficult to repeat [29, 30]. Hence, the development of an efficient transient gene expression system that could be exploited to characterize gene functions and investigate molecular processes in cassava is required.

Due to the fact that cassava is recalcitrant for plant regeneration from protoplasts, only two reports about the protoplast culture and regeneration of cassava have been published. Shahin and Shephard reported shoot regeneration from the isolated mesophyll protoplasts of cassava [31] and Sofiari et al. reported the plant regeneration from protoplasts isolated from friable embryogenic callus of cassava [32]. Anthony et al. developed a protocol for the culture of cassava leaf protoplasts but plant regeneration from protoplasts had not achieved [33]. To date, transient gene expression in cassava protoplasts has not been developed. Here we report an effective and reliable mesophyll protoplast isolation and PEG-mediated transient gene expression in cassava. The protocol has been successfully used for subcellular localization of a sugar transporter, MeSTP7 protein.

## Methods

### Materials

*In vitro* plantlets of *Manihot esculenta* cv. South China 8 (SC8) were used in this study. Cultures were kept on 1/2MS (Murashige and Skoog) medium (supplemented with 2% sucrose and 0.8% agarose, pH5.8) at 28 °C, under lighting with a cycle of 12 h/8 h (light/darkness) for 6-10 weeks to obtain fully expanded leaves.

### Protoplast isolation

Protoplast isolation was conducted using the protocols described by Yoo et al. [3] and Anthony et al. [33] with slight modification. The fully expanded green leaves were cut into about 0.5-1.0 mm strips with sharp razors. The strips of 0.3 ± 0.03 g (for each treatment) were transferred quickly into 10 mL of enzyme solution (0.1% BSA, 9% mannitol, 20 mM KCl, 10 mM CaCl_2_, 20 mM MES, pH 5.8) with different concentrations of cellulase R-10 (0.8%, 1.6% or 2.4%) (Yakult, Japan) and macerozyme R-10 (0.4%, 0.8% or 1.2%) (Yakult, Japan) for cell wall hydrolysis by shaking at 45 rpm for 16 h in the dark (25 °C) for plasmolysis.

The digested tissues were filtered through a 0.75-mm nylon sieve, collected by centrifugation (80 *g* for 3 min) and suspended in 20 mL pre-cooled W5 solution (154 mM NaCl, 125 mM CaCl_2_, 5 mM KCl and 2 mM MES, pH 5.8). The protoplast pellet was purified twice in W5 solution by repeated resuspension and centrifugation (80 *g* for 3 min).

The resulting protoplasts were resuspended in 2 mL W5 solution, placed on the ice for 30 min and counted under a microscope equipped with a hemocytometer. The viability of protoplasts was measured with 0.2% fluorescein diacetate (FDA) staining and determined as follows: protoplast viability (%) = (fluorescent protoplast number in view/protoplast total number in view) × 100%.

### Cassava mesophyll protoplast transformation

The protoplasts were collected by centrifugation (80 *g* for 2 min) and resuspended in MMg solution (9% mannitol, 15 mM MgCl_2_, 4 mM MES) to a density of 1.0 × 10^7^ protoplasts/ml. 15 μg of pA7-GFP plasmid [34] were mixed gently with 200 μl protoplasts. Then different concentrations of freshly prepared PEG4000 solutions (9% mannitol, 100 mM CaCl_2_) were immediately added and mixed by gentle inversion to obtain a 15%, 20%, 25% or 30% final PEG4000 concentration. The mixture was incubated at room temperature for 5, 10, 15 and 20 min, respectively. After incubation, the transfection mixture was gently diluted with three volumes of W5 solution.

The transfected protoplasts were centrifuged (80 *g*, 2 min) twice, then resuspended with 300-500 μl WI solution (4 mM MES, 9% mannitol, 20 mM KCl, pH 5.8) and incubated overnight at room temperature in dark for the induction of protein expression. Transformation efficiency was detected under fluorescence microscopy and the expression of GFP tag was observed by confocal laser scanning microscope. Transformation efficiency was calculated as follows: transformation efficiency (%) = (bright green fluorescent protoplast number in view/total protoplast number in view).

### Plasmid construction and subcellular localization of MeSTP7 protein

Total RNA was extracted from cassava leaves with RNAplant Plus (Tiangen biotech, Beijing, China) and cDNA was synthesized using the PrimeScript RT reagent Kit with gDNA Eraser (TaKaRa, Dalian, China) following the manufacturer’s instructions.

The specific primers with *Xho* I and *Spe* I restriction enzyme sites (bold) introduced to their ends respectively, STP7-F (5'-CCG**CTCGAG**ATGCCTGCAGGAGGTTT-3') and STP7-R (5'-GG**ACTAGT**GAAAACTGGGTAACAGGATCAA-3'), were designed according to the conserved region of *MeSTP7* (GenBank accession number: Manes.03G180400). The gene of interest was amplified using Phusion DNA Polymerase under the following conditions: 98 °C for 30 s followed by 35 cycles of amplification (98 °C for 10 s, 57 °C for 30 s, 72 °C for 45 s) and a 10-min final extension at 72 °C.

**Fig. 1:**
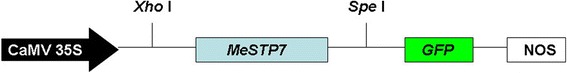
Schematic representation of the *pA7-MeSTP7-GFP* construct driven by the constitutive CaMV 35S promoter and terminated by nopaline synthase terminator (NOS)

The PCR products were confirmed by sequencing and inserted between the 35S promoter and the *GFP* of the expression vector *pA7-GFP*, designated as *pA7-MeSTP7-GFP* (Fig. 1). The plasmid construct was propagated in competent *Escherichia coli* Trans-T1 (TransGen Biotech, Beijing, China). Subcellular localizations of MeSTP7 protein in cassava and *Arabidopsis* mesophyll protoplasts were carried out according to our transformation procedure described above and the method established by Yoo et al [3], respectively.

## Results

### Isolation of protoplasts from cassava leaves

**Fig. 2:**
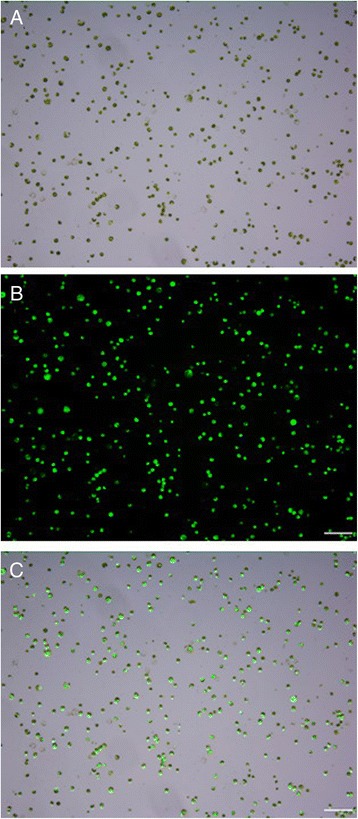
The viability assays of cassava mesophyll protoplasts using the optimized protocol, i.e. preparation of mesophyll protoplasts in an enzyme solution (1.6% cellulase + 0.8% macerozyme) incubated at 25 °C (45 rpm) for 16 h in darkness. **a** Bright field image of protoplasts; (**b**) Protoplasts stained with FDA; (**c**) Merged image of protoplasts (Scale bar = 100 μm).

Different enzyme combinations significantly influenced the yield and viability of cassava protoplasts. Protoplast yield increased with increasing concentrations of cellulase R-10 and macerozyme R-10, but protoplast viability decreased with increasing enzyme concentrations. It was found that the highest production of protoplasts (5.1 × 10^7^ protoplasts/ g FW) was obtained with 2.4% cellulose and 1.2% macrerozyme, with the lowest viability of protoplasts (83.8%). For obtaining the highest number viable protoplasts for the subsequent transfection experiments, the optimum combination of enzymes for cassava protoplast isolation was determined as 1.6% cellulase + 0.8% macerozyme. This combination gave the yield of 4.4 × 10^7^ protoplasts/g FW and the viability of 92.6%. The enzyme solution turned green, indicating the release of mesophyll protoplasts. Leaf mesophyll protoplasts were uniformly spherical, and containing single intact nucleus and green chloroplasts. (Table 1, Fig. 2).

**Table 1:** Effects of enzyme combinations on the yield and viability of protoplasts isolated from leaves of cassava

Cellulase R-10 (*W/V*)	Macerozyme R-10 (*W/V*)	Protoplast yield (10^7^#/g FW)	Protoplast viability (%)
0.8	0.4	1.08 ± 0.14f	93.64 ± 1.00a
0.8	0.8	1.45 ± 0.09ef	93.05 ± 3.00a
0.8	1.2	1.89 ± 0.14e	91.32 ± 1.00a
1.6	0.4	3.08 ± 0.14d	93.43 ± 2.00a
1.6	0.8	4.42 ± 0.29b	92.60 ± 2.00a
1.6	1.2	3.25 ± 0.39 cd	90.14 ± 3.00a
2.4	0.4	1.80 ± 0.15e	88.38 ± 4.00ab
2.4	0.8	3.66 ± 0.18c	87.67 ± 1.00ab
2.4	1.2	5.06 ± 0.25a	83.82 ± 5.00b

Note: The averages of three technical replicates ± standard error of the mean (SEM) are shown. The different letters indicate significant differences (P ≤ 0.05) according to the Duncan test

### Establishment of cassava mesophyll protoplast expression system

**Fig. 3:**
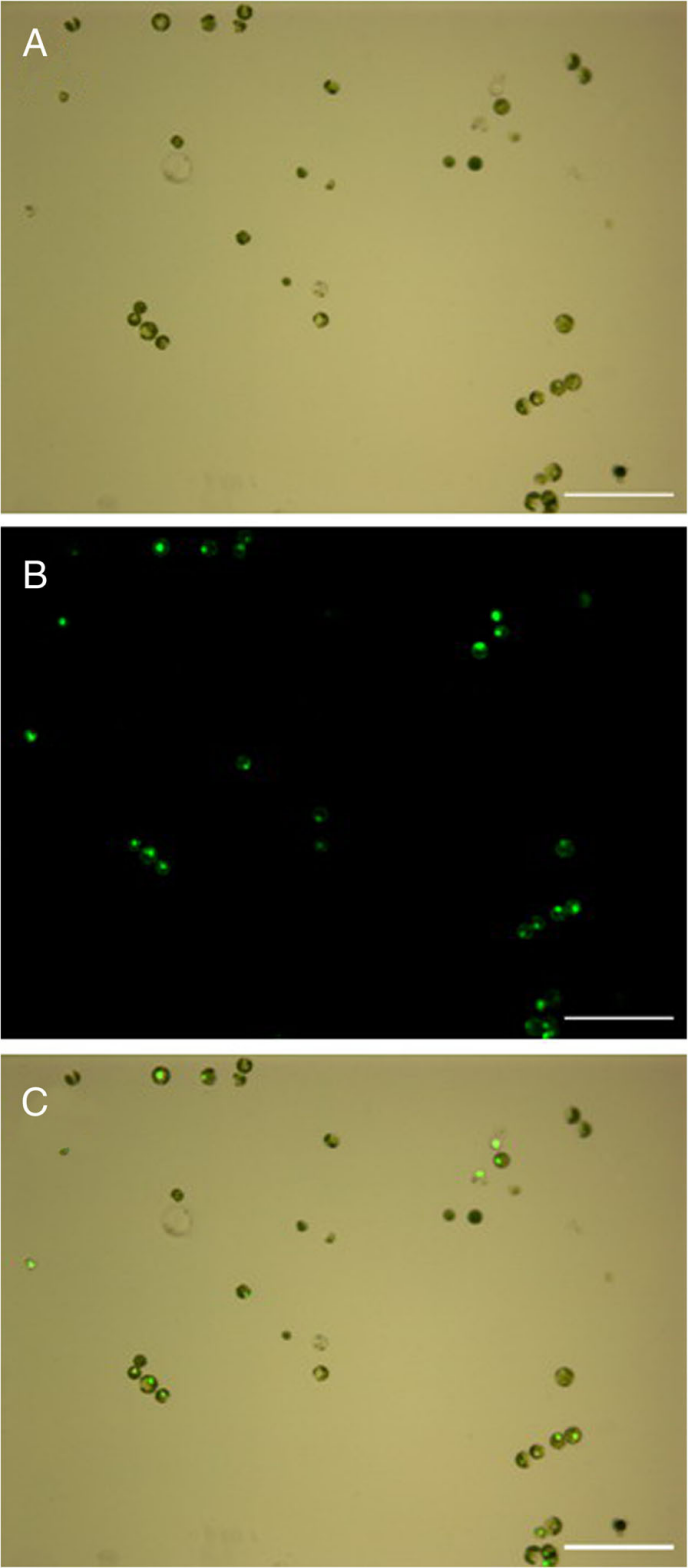
High-efficiency transformation of cassava mesophyll protoplasts with pA7-GFP plasmid. **a** Bright field image of protoplasts; (**b**) Image of GFP; (**c**) Image of GFP merged with chlorophyll autofluorescence (Scale bar = 100 μm)

**Fig. 4:**
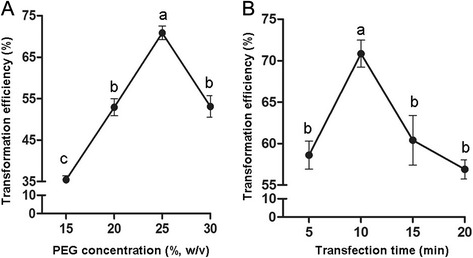
Effects of PEG4000 concentration (**a**) and transfection time (**b**) on cassava protoplast transformation efficiency. Values are expressed as mean ± SEM, the error bars represent at least five independent replicates. The different letters indicate significant differences (P ≤ 0.05) according to the Duncan test

In this study, the effects of PEG4000 concentration and transfection duration on cassava protoplast transformation efficiency was investigated with the transient expression vector *pA7-GFP*. The results showed that when the transfection time was 10 min, the transfection efficiency increased along the increasing concentration of PEG4000 and reached the maximum level (70.8%) at the concentration of 25% (Figs. 3 and 4a). When the concentration of PEG4000 was 25%, transfection efficiency increased with increases in the transfection time until it peaked at 10 min (Figs. 3 and 4b). Therefore, factors affecting transfection efficiency were optimized: 25% of PEG4000 concentration and 10 min of transfection time. In addition, stability of transient expression of GFP has been verified as GFP signal could be detected 8-30 h after transformation.

### Subcellular localization of GFP-fused MeSTP1 protein in cassava mesophyll protoplasts

**Fig. 5:**
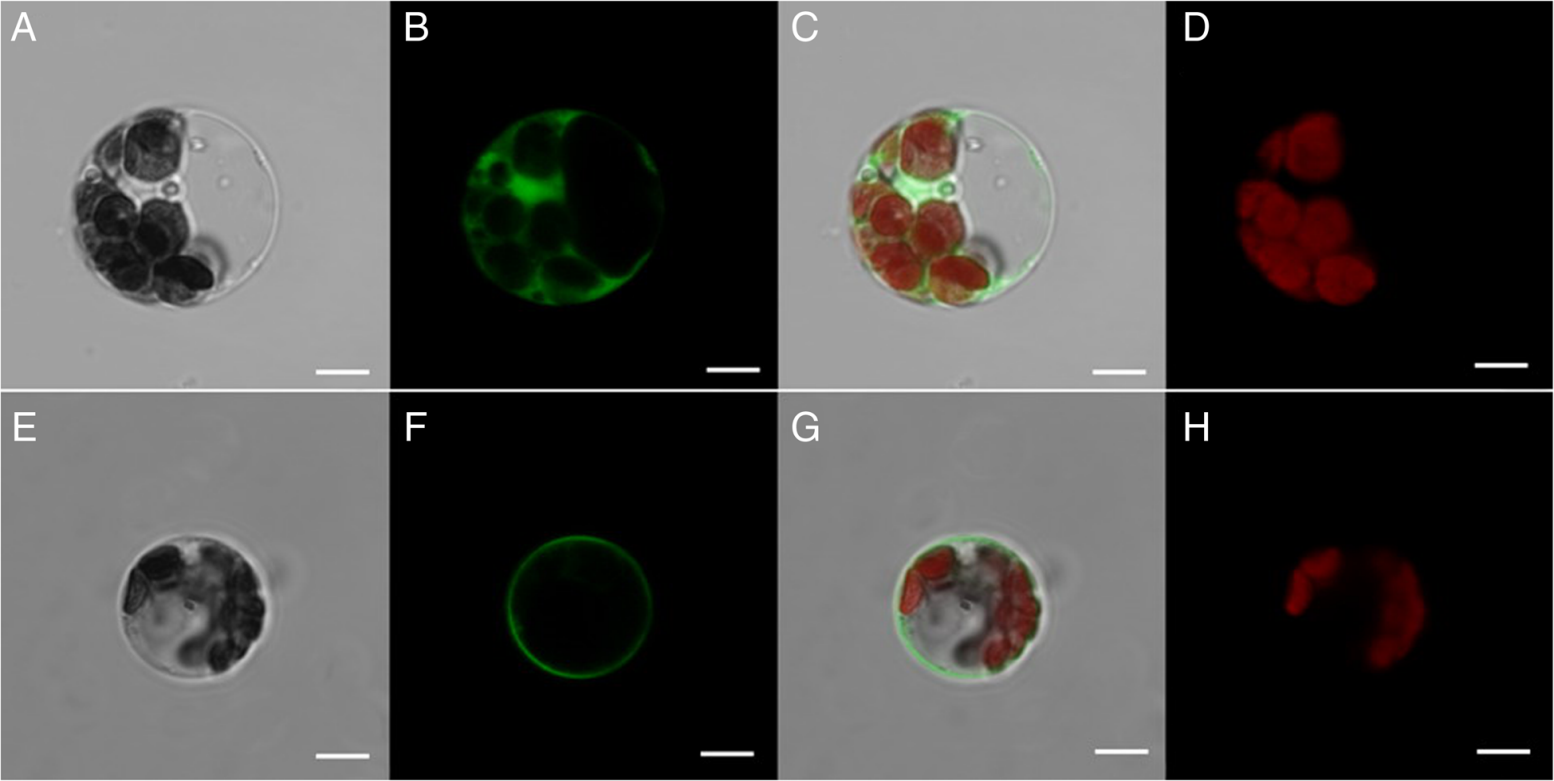
Subcellular localization of MeSTP7 in cassava mesophyll protoplasts. Transient expression of GFP, showing that the GFP is distributed throughout the nucleus and cytoplasm (**a**-**d**). The laser-scanning confocal microscopy images are the bright field image (**a**), fluorescence image (**b**), merged image (**c**) and autofluorescence image (**d**), respectively. The transient expression of GFP-fused MeSTP7 protein, showing that the MeSTP7-GFP fusion protein is likely localized to plasma membrane (**e**-**h**). The laser-scanning confocal microscopy images are the bright field image (**e**), fluorescence image (**f**), merged image (**g**) and chloroplast fluorescence (H), respectively. The bars = 5 μm.)

**Fig. 6:**
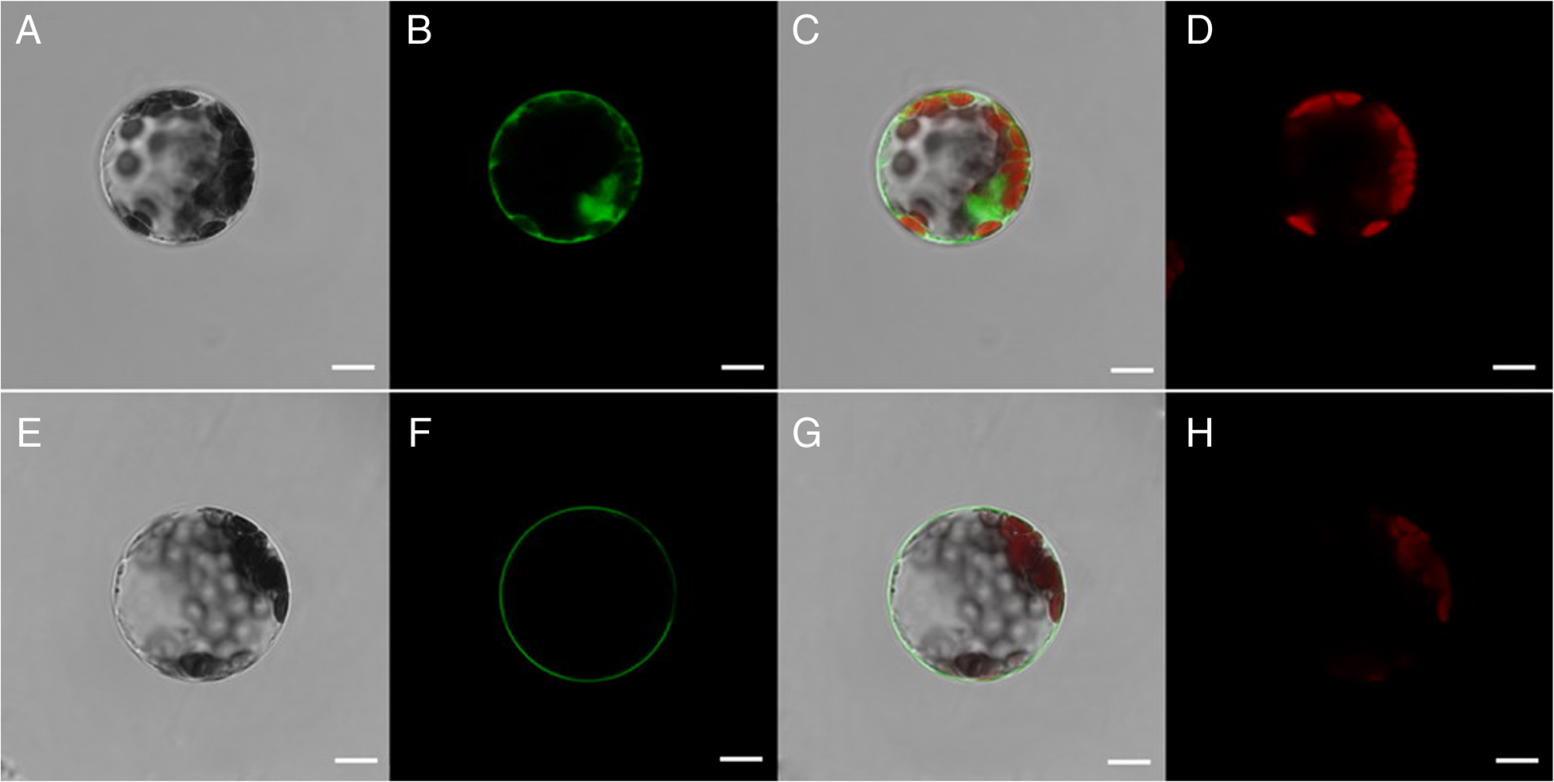
Subcellular localization analysis of MeSTP7 in *Arabidopsis* mesophyll protoplasts. Transient expression of GFP, showing that the GFP is distributed throughout the nucleus and cytoplasm (**a**-**d**). The laser-scanning confocal microscopy images are the bright field image (**a**), fluorescence image (**b**), merged image (**c**) and chloroplast fluorescence image (**d**), respectively. The transient expression of GFP-fused MeSTP7 protein, showing that the MeSTP7-GFP fusion protein is likely localized to cytoplasm membrane (**e**-**h**). The laser-scanning confocal microscopy images are the bright field image (**e**), fluorescence image (**f**), merged image (**g**) and chloroplast fluorescence image (**h**), respectively. The bars = 10 μm

To test the feasibility of the protoplast transient expression system for the subcellular localization of protein in cassava cells, the transient expression of GFP-fused MeSTP7 protein in cassava mesophyll protoplasts was performed. Twelve-eighteen hours after transfection, brilliant green fluorescence distributed in intracellular compartments was observed. MeSTP7:GFP was specifically detected in cell membrane, but empty vector control (GFP) was distributed throughout the nucleus and cytoplasm (Fig. 5). Similar pattern of MeSTP7 subcellular localization in *Arabidopsis* mesophyll protoplasts validated the authenticity of the results described above (Fig. 6).

## Discussion

A reliable protoplast isolation with high yield and viability is prerequisite for the success of transient gene expression. Source tissue, the type and concentration of enzymes are critical factors affecting the release of protoplasts [3, 35, 36]. Leaves from field or greenhouse-grown plants usually have a thick cuticle and recalcitrant cell walls, which are likely to be the major limiting factors for protoplast isolation [12]. In cassava, Shahin et al. [31] used well expanded leaves which were collected from preconditioned plants (in growth chamber) and then incubated on a solution supplemented with naphthaleneacetic acid (NAA) and benzylaminopurine (BAP) as protoplast source. Sofiari et al. [32] isolated cassava protoplasts from friable embryogenic callus (FEC) and from suspensions derived from FEC. Anthony et al. [33] took juvenile leaves from axenic shoot cultures of cassava as protoplast source. For convenience sake, we chose the leaves from *in vitro* propagated cassava plants as the protoplast source.

For a given species and source tissue, suitable enzymolysis time and enzymolysis composition are vital for protoplast isolation. Anthony et al. [33] had established an improved protocol for cassava protoplast isolation with an enzyme mixture for 16 h. Referring to the method described by Anthony et al. [33], optimization of the enzymolysis composition was conducted in this study. The combinations of cellulase and macerozyme which have been commonly used for protoplast isolation, were evaluated to obtain protoplasts with the highest yield and viability. As the concentration of total enzyme in the mixture increased, protoplast yield seemed to increase but protoplast viability to decrease. High levels of cellulose R-10 and macrerozyme R-10 increased protoplast yield but decreased the viability of protoplasts, possibly due to the influence of enzymes on the integrity of membrane and the physiological activities of protoplasts [37, 38]. The highest yield (5.1 × 10^7^ protoplasts/ g FW) and the lowest viability of protoplasts (83.8%) were recorded with the 2.4% cellulose and 1.2% macrerozyme, which was the most concentrated enzyme mixture in our study. This lowest viability of protoplasts may not be suitable for subsequent experiments. However, the lowest concentrations of enzymes in the isolation mixture gave a lower yield (1.8 × 10^7^ protoplasts/g FW) but with a slightly higher viability of protoplasts (93.6%). The enzyme mixture containing 1.6% Cellulase R10 and 0.8% macerozyme R10 was the best mixture in terms of both yield (4.4 × 10^7^ protoplasts/g FW) and viability (92.6%) of protoplasts. The maximum yield and viability of leaf mesophyll protoplasts achieved in our study were substantially higher than the results previously reported [28, 30]. The reason possibly attributed to the differences in tissue source used [31, 32], and/or the type and concentration of enzymes [32, 33]. In addition, the high yield (4.4 × 10^7^ protoplasts/g FW) and vitality (92.6%) of cassava mesophyll protoplasts obtained in our study were comparable to the values reported for *Populus* (1 × 10^7^ protoplasts/g FW, more than 90%) [13], *Brachypodium distachyon* (1.7 × 10^7^ protoplasts/g FW, more than 90%) [19], cucumber (6-7 × 10^7^ protoplasts/g FW, about 90%) [15]. The high yield and viability allowed the application of this system for the subsequent transfection experiment.

Although heterologous transient gene expression systems with *Arabidopsis* and tobacco protoplasts are commonly adopted for studying gene function in non-model plants such as cassava, these heterologous systems may display aberrant results due to a foreign genetic background. In this study, we established an efficient cassava protoplast transfection system by optimizing PEG4000 concentration and transfection time. This protocol will be an extraordinarily valuable tool for cassava gene functional study. In addition, transfection efficiency over 50% is generally required for obtaining reliable and reproducible results with the protoplast system [3]. The highest transfection efficiency (70.84%) with strong GFP signal was obtained with 25% of PEG4000 concentration for 10 min of transfection time. This result demonstrated that the transfection efficiency of cassava protoplasts was sufficiently high to be used for gene functional analysis in the future.

Using the optimized protocol that we developed, subcellular localization of MeSTP7 was investigated. Cassava *MeSTP7* is a homologous gene of *STP1* (*SUGAR TRANSPORTER PROTEIN 1*) in *Arabidopsis thaliana* and *Nicotiana tabacum*, which encodes an H^+^/monosaccharide cotransporter containing 12 putative transmembrane domains and is a putative plasma membrane protein [39–41]. Our results showed that MeSTP7 have been likely localized to the cassava plasma membrane. Similar to the localization in cassava protoplasts, the MeSTP7 protein was distributed in *Arabidopsis* plasma membrane using a heterologous transient gene expression system. This confirmed that the cassava mesophyll protoplast expression is a reliable procedure for subcellular protein localization.

## Conclusions

In summary, a highly efficient protocol for cassava mesophyll protoplast isolation and PEG-mediated transient expression was obtained through optimization of the type and concentration of enzymes for protoplast preparation, and PEG4000 concentration and treatment time for transfection. The method reported here can thus be very useful for molecular and cellular studies in cassava, especially combined with genetics, genomics, transcriptomics and proteomics. It is noteworthy that the simple incubation buffers described in this article do not support division of mesophyll protoplasts. Therefore, the system will not appropriate to evaluate the expression of cassava genes involved in, e.g., cell cycle, plant dedifferentiation, or genes that require long-term follow-up (several cycles of cell division). The protoplast regeneration protocols developed by Shahin and Shephard [31], Sofiari et al [32] and Anthony et al [33] can be used for this purpose.
